# Remarks on Mr. Walker's Journal of the Progress of Small-Pox

**Published:** 1816-08

**Authors:** James Woodham


*For the London Medical and Physical Journal.*

### *Remarks on Mr. Walker's Journal of the Progress of Small-pox;* by JAMES WOODHAM, Esq.

IF quantity and not quality be a criterion of merit, the papers of Mr. Walker must be allowed to be pre-eminently praise-worthy; but, if the converse be the test, it would be a moot point I believe with the majority of your readers. For in the whole of those essays, somewhat "lengthy," according to the elegant phraseology of the Americans, with which he has favored the public, through the medium of the Medical and Physical Journal, I recollect only only one practical fact but what must have been familiar to almost every apothecary's apprentice in the kingdom. They have been mere enumerations of common-place every-day occurrences, not worth recording, not worth perusing. Under this sweeping censure, as it may be termed, I also include his last extraordinary communication, and which, if "*Nil admirari*" had not long since been my motto, I should have felt a considerable degree of astonishment that, in this æra of medical science, and when vaccination was spreading over the four quarters of the globe, a grave practitioner should have thought it necessary to publish a journal of the progress and treatment of *small-pox!!!*

I believe it will be found, that, in every system and compendium of medical practice for these last forty years, ending with Dr. Thomas's Modern Practice of Physic, ample and correct directions are given for the conducting of patients labouring under small-pox, as well as an enumeration of its phenomena or symptoms. Admitting then the premises of Mr. Walker, which some may deem questionable, yet his conclusion by no means follows. More *Vade-mecums* (more last words of Mr. Partridge) are not wanted, the press groans under them, and the market is completely glutted, as may be seen by a cursory inspection of any medical bookseller's catalogue. Mr. Walker's journal therefore is a work of supererogation, useless and uncalled for.

From the circumstances then of the diffusion of vaccination, the general decline of small-pox, and the multiplicity of books already on the subject, I cannot but think that something more is meant than meets the eye in the journal of Mr. Walker. Under the specious pretext of improving the treatment of a disease growing daily less and less common, a blow is indirectly aimed, I conceive, at one of those great blessings which the Creator has bestowed on us. Vaccination, as we are informed by Mr. Walker in his multifarious contributions to the Medical and Physical Journal, has been somewhat unsuccessful in the circle in which he moves; but it is surely bad logic, and excusable only in a young practitioner, to generalize from too few facts; and those which have fallen under Mr. Walker's observation must have been few comparatively. He may say, in the words of the poet,—

"What can we reason, but from what we know?"

Granted; but it should be, not what we know from our own limited experience alone, but from the experience of others conjoined with our own. This is to reason justly and philosophically.

Vaccination, however, will, I am persuaded, proceed in spite of the ignorant and the uninformed, the interested and the prejudiced; and, when I am told of its failures, of its not being a permanent security, the cause of cutaneous eruptions, and such *fabulæ aniles*, I reply in the words of Franklin, who, when ambassador at the court of France during the American revolution, on being told that his countrymen had suffered a defeat, answered " *n'importe c'ira.*"

*London;*
*July* 6, 1816.

---

